# Supplementary material for: Effects of Various Ripening Media on the Mesoporous Structure and Morphology of Hydroxyapatite Powders
Source: Nanomaterials (Basel). 2023 Jan 19;13(3):418. doi: 10.3390/nano13030418 (PMC9919035; doi:10.3390/nano13030418)
Supplement: Supplementary file 1 [file nanomaterials-13-00418-s001.zip › nanomaterials-2109918-supplementary.pdf]

Supplementary Materials

Effects of Various Ripening Media on the Mesoporous Structure and Morphology of Hydroxyapatite Powders

Margarita A. Goldberg [1,*], Olga S. Antonova [1], Nadezhda O. Donskaya [1], Alexander S. Fomin [1], Fadis F. Murzakhanov [2], Marat R. Gafurov [2], Anatoliy A. Konovalov [1], Artem A. Kotyakov [1], Alexander V. Leonov [3], Sergey V. Smirnov [1], Tatiana O. Obolkina [1], Egor A. Kudryavtsev [4], Sergey M. Barinov [1] and Vladimir S. Komlev [1]

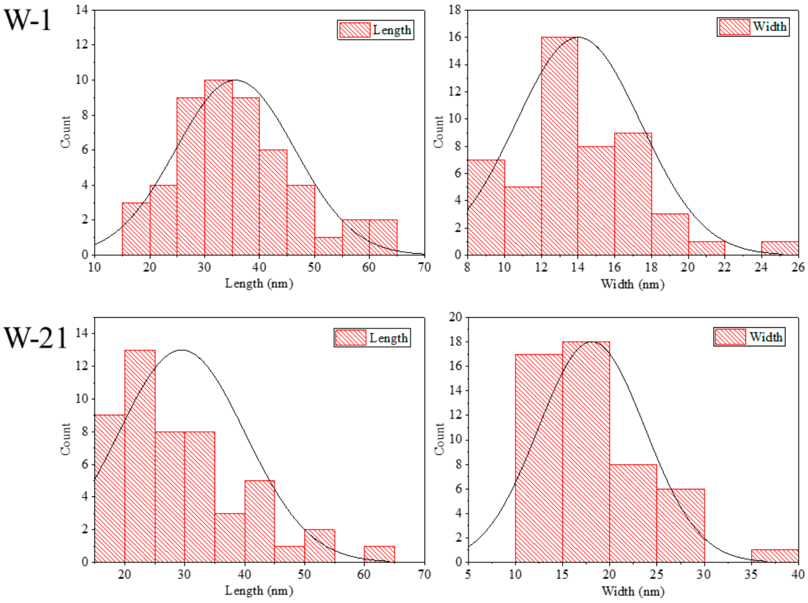

(a)

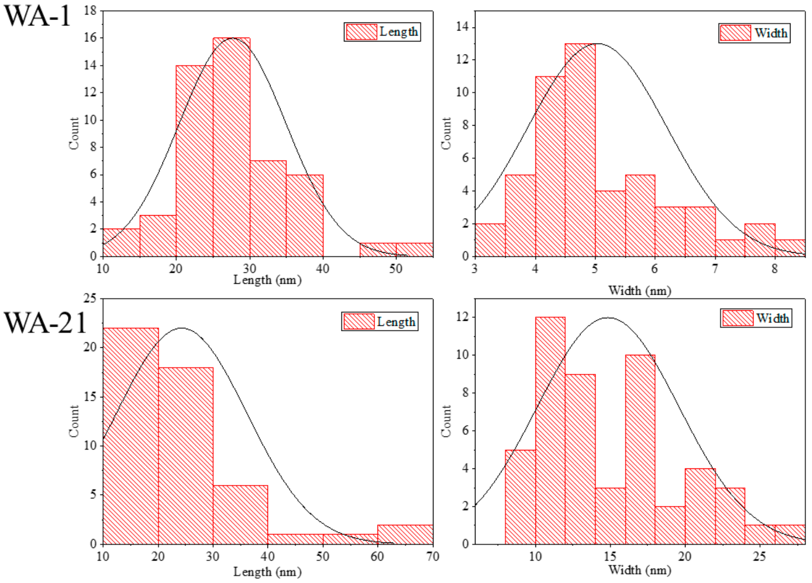

(b)

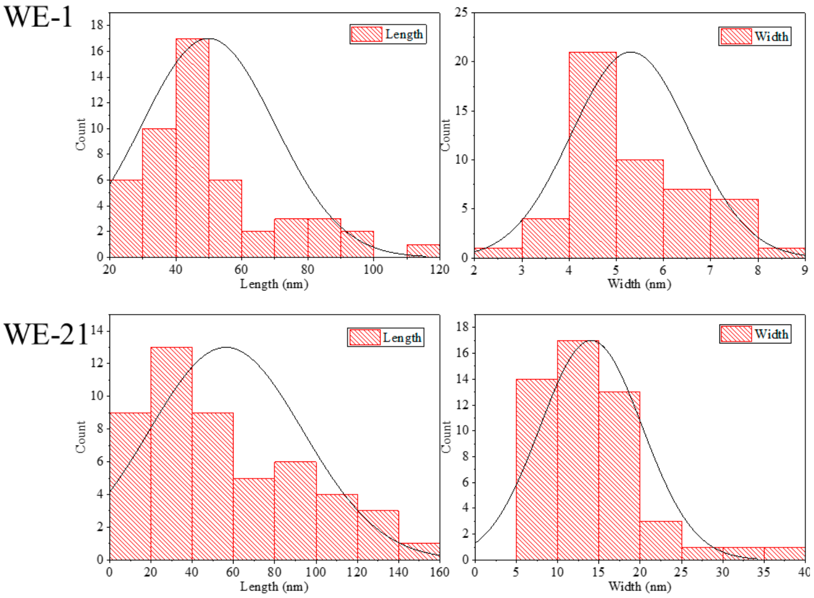

(**c**)

Figure S1. Particle length and width distribution according to TEM data, there (a)- water-synthesized materials, (b) – a water-acetone mixture, (c) – a water-ethanol mixture.
